# Diagnostic roles of proliferative markers in pathological Grade of T1 Urothelial Bladder Cancer

**DOI:** 10.7150/jca.52336

**Published:** 2021-03-05

**Authors:** Jianping Yang, Chunjun Li, Yong Tang, Fang Guo, Yu Chen, Wenqi Luo, Xiaoyu Chen, Yun Ma, Lixia Zeng

**Affiliations:** 1 Department of Pathology, Guangxi Medical University Cancer Hospital, Nanning 530021, Guangxi Zhuang Autonomous Region, China.; 2 Department of Urology, Wuming Hospital of Guangxi Medical University, Nanning 530199, Guangxi Zhuang Autonomous Region, China.; 3 Department of Pathology, Hubei Cancer Hospital, Tongji Medical College, Huazhong University of Science and Technology, Wuhan 430070, Hubei, China.

**Keywords:** urothelial bladder cancer, telomerase reverse transcriptase promoter mutations, mitotic index, Ki-67

## Abstract

The stage T1 urothelial bladder cancer (T1 UBC) tumor grade classification is important for prognosis and clinical management. However, the reproducibility of this two-grade classification system is limited in regards to pathological diagnosis, and there is lack of ideal, objective and easily detected markers for pathological diagnosis. In our study, bladder urothelial lesions from a total of 124 patients diagnosed pathologically after transurethral resection of the bladder tumor (TURBT) were collected, including non-cancerous lesions from 33 patients and lesions from 91 T1 UBC patients. A series of previous studies have suggested some common and valuable factors in the diagnosis and prognosis of UBC, but there are still some controversial factors, such as the mitotic figure (MF) of tumor cell, cell proliferation index Ki-67, graded differentiation marker CK20, P53, P504S and carcinogenesis associated telomerase reverse transcriptase (TERT) promoter mutations. The purpose of this study was to evaluate the value of these factors in the pathological grading diagnosis of T1 UBC. The results showed that gender, lesion size, mitotic index (MI), CK20, P53, Ki-67, P504S and TERT promoter hot spot mutations (C228T and C250T) were correlated with T1 UBC diagnosis (P<0.05). The MI, Ki-67 and P504S were correlated with the pathological grade of T1 UBC (P<0.05). Logistic regression analysis showed that the MI and Ki-67 were independent risk factors for high-grade (HG) of T1 UBC (P<0.05). The combined detection of the MI, Ki-67 and P504S in a multivariate diagnostic model improved the diagnostic accuracy of assigning the T1 UBC pathological grade (AUC=0.904, 95%CI: 0.824~0.956, P<0.05). In conclusion, MI and Ki-67, as important markers of histopathology and cell proliferation, can be easily measured and have good reproducibility. These markers may be meaningful parameters for assigning the pathological grade of UBC.

## Introduction

Urothelial bladder cancer (UBC) is a common malignant tumor. UBC is more common in men than in women. Recently, the incidence of UBC in China has been increasing annually 1. Approximately 75% of patients are found to have non-muscle-invasive bladder cancer (NMIBC) at the time of their first diagnosis, of which approximately 20% are T1 stage bladder cancer 2. Transurethral resection of bladder tumor (TURBT) with or without adjuvant therapy is the standard therapy for NMIBC. To different stages and grades of NMIBC, the treatment or management vary in principle 3, 4. For example, intravesical chemotherapy is often performed for Ta low-grade (LG) UBC, and the National Comprehensive Cancer Network (NCCN) guidelines recommend a second TURBT combined with Bacillus Calmette-Guérin (BCG) immunotherapy as the preferred treatment for T1 LG UBC. To Ta high-grade (HG) UBC, BCG immunotherapy or chemotherapy should be supplemented after surgery, and TURBT would be considered again according to whether it is completely removed. Early radical cystectomy should be performed for T1 HG UBC. If the pathological grade of T1 UBC cannot be correctly distinguished, it may lead to excessive or improper treatment. The latest research suggests that the NCCN guidelines are simple and practical for predicting tumor recurrence and progression. It can be used as the first choice 5. The NCCN further emphasizes the importance of UBC tumor grading for prognosis and treatment decisions.

The WHO classification of tumors of the urinary system and male genital organs (2004 and 2016 version) divided UBC into two grades: high and low 6. However, due to the histology diversity of UBC and the lack of a defined threshold between high-grade (HG) and low-grade (LG) tumors, the diagnostic reproducibility of the two-stage classification system is limited 7, which may lead to deviations in treatment decision-making, namely excessive or improper treatment. The clinical standardized treatment strategy is facing severe challenges, and it is urgent to optimize a diagnostic and individualized prognostic risk assessment model. Molecular analysis shows a high degree of genomic imbalance in pT1 UBC compared with NMIBC 8. The clinical outcome of pT1 UBC is also significantly different, especially in some patients with pT1G3, which can rapidly progress to muscle-invasion and metastasis 9. The prognosis of patients in pT1 UBC is significantly different. Stratification analysis is important for diagnosis, treatment and follow-up strategies in UBC research. In order to better control the influence of confounding factors on the results, it is necessary to separate T1 UBC from NMIBC as an independent research object. Ideal molecular markers for application of clinical diagnosis should be convenient, quick, objective and economical. At present, there are few studies on the diagnostic and prognostic evaluation combined with histopathologic features and molecular pathological markers of T1 UBC. Although the WHO criteria on the histopathology of UBC mentioned that the mitotic figure (MF) was related to tumor grade, but mitotic count is not featured prominently, and no specific cut-off is presented 7.

Previous studies have suggested that some markers are valuable in the diagnosis and prognosis of UBC, but there are still controversial results, such as those regarding the cell proliferation index Ki-67 10, graded differentiation marker CK20 11, P53 12, P504S 13 and carcinogenesis associated telomerase reverse transcriptase (TERT) promoter mutations 14, combined with the UBC mitotic index (MI). In this study, these markers were evaluated in regards to their diagnostic value in predicting T1 UBC pathological grading.

## Materials and Methods

### Patient selection

From January 2016 to December 2017 patients who were biopsied for the first time by TURBT at the Department of Pathology, Cancer Hospital of Guangxi Medical University, were included. There were 124 cases of bladder urothelial lesions diagnosed pathologically that had negative clinical examination margins. The tissue sections of this study sample were retrospectively analysed, according to the 2016 WHO classification of bladder cancer, by two pathologists with extensive experience in diagnosing urinary system diseases. The staging of the tumors follows the 8^th^ edition of the Union for International Cancer Control/American Joint Committee on Cancer (UICC/AJCC), and stage T1 bladder cancer refers to the invasion of the tumor into the subepithelial connective tissue but not yet invaded the muscularis propria. After the operation, the leisions were not complicated with CIS, or the patients' wishes were followed in regards not to early radical cystectomy. With the approval of the Hospital Ethics Committee, the clinical data were retrospectively collected. The clinical data included medical records, re-examination records and telephone follow-up.

The isolated specimens of TURBT were fixed with 10% neutral formalin, dehydrated, embedded in paraffin, cut into sections for hematoxylin-eosin staining (HE) and observed under a microscope.

### Mitotic figure count and mitotic index (MI)

**Figure 1:**
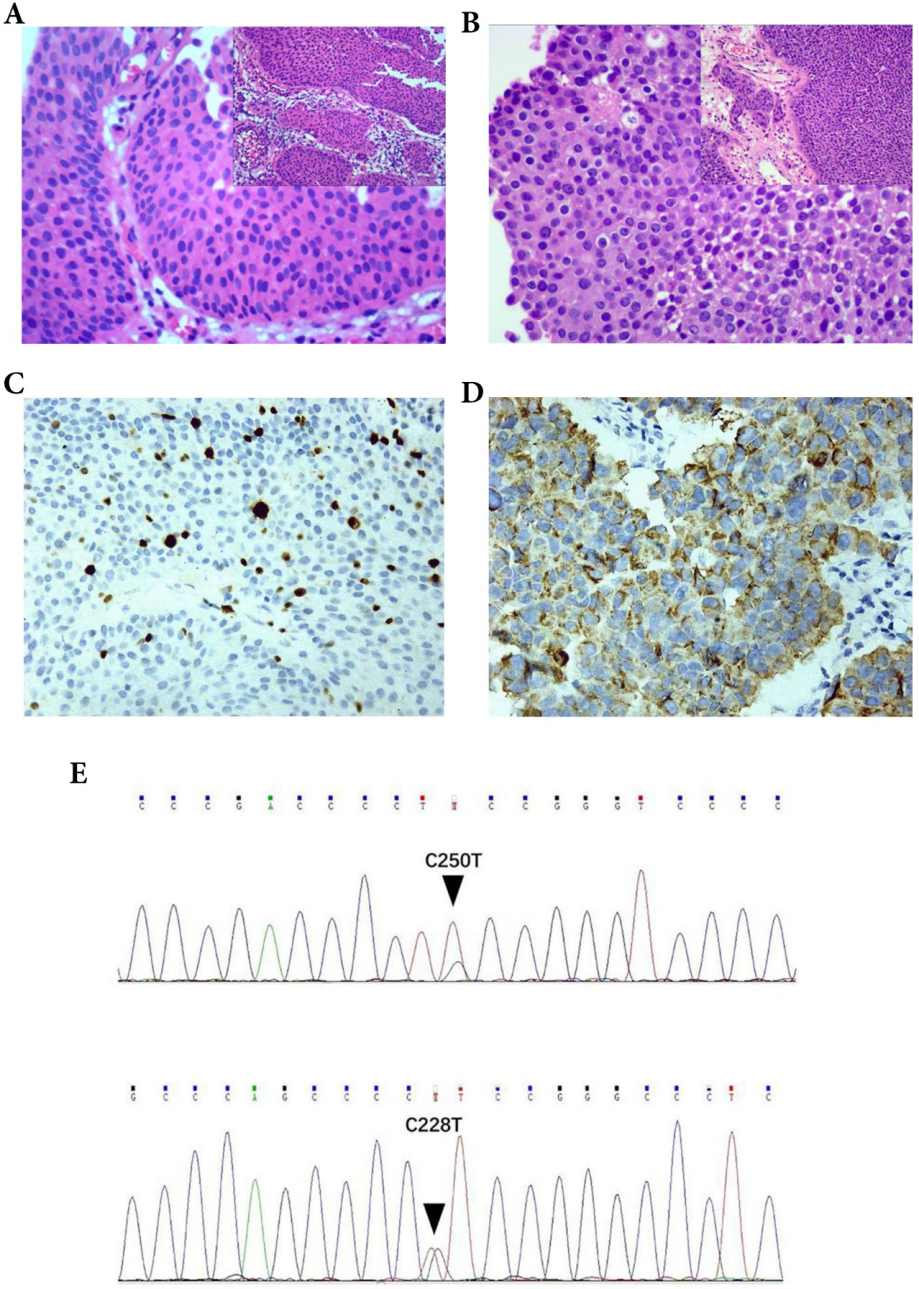
The parameters were correlated with the pathological diagnosis of T1 UBC. The mitotic index (MI) in T1 UBC A. MI ≤ 2 (200×), B. MI > 2 (200×). Expression of Ki-67and P504S in immunohistochemistry (IHC) C. Ki-67, D. P504S. Hot spot mutations of the TERT promoter in UBC (C250T and C228T) E.

The HE sections of the 124 cases of bladder urothelial lesions were observed under a microscope. The mitotic figures were counted at an objective magnification of 400×. Its field diameter is 0.55 mm and field area is 0.2376 mm^2^ per high power visual field (HPF 400×). The most active areas of MF in the tumor tissues were selected, and at least 5 high power visual fields (HPF 400×) were counted. The mitotic index (MI) (Figure 1A-B) was calculated as the average number of mitoses per high power visual field (HPF 400×, 0.2376 mm^2^). MI is a quantitative mitotic frequency analysis method which combines the number of mitotic figures with square millimeter (mm^2^) of neoplastic epithelium. In our research, we used a threshold of MI set to 2/HPF (field area: 0.2376 mm^2^) nearly 10/mm^2^, such as MI ≤ 2/HPF (- or mitotic-inert) and > 2/HPF (+ or mitotic-active) 15-17 (Fig. 1A, B).

### Immunohistochemical staining and evaluation

CK20 (mouse anti-human monoclonal antibody: Ks20.8), P504S (rabbit anti-human monoclonal antibody: 13H4), P53 (mouse anti-human monoclonal antibody: MX008) and Ki-67 (mouse anti-human monoclonal antibody: MIB-1) were purchased from Maixin Biotechnology Co., Ltd, Fujian, China. The protein expression of CK20, P504S, P53 and Ki-67 was detected by a Super-Vision two-step method. After routine dewaxing and hydration, antigen repair was completed according to the instructions of the kit. Then the slides were incubated with the first antibody and the second antibody, stained with diaminobenzidine (DAB), restained with hematoxylin and then sealed and observed.

CK20 and P504S (Fig. 1D) were observed in the cytoplasm, while P53 and Ki-67 (Fig. 1C) were observed in the nucleus. The threshold selection of the positive expression of each index is detailed in Table 1.

### Detection of TERT promoter mutations

Tissue sections were reviewed by pathologists to confirm the histopathologic diagnosis and ensure that at least 50% of the cells used for DNA extraction were neoplastic. Genomic DNA was extracted from typical tumor areas that were scraped from formalin-fixed and paraffin-embedded tissue slides as previously described. Genomic DNA from the tumor tissues was isolated using the standard procedures of the QIAamp DNA FFPE Tissue Kit (QIAGEN, Hilden, Germany). The DNA concentration and purity were measured by a ND8000 spectrophotometer (NanoDrop Technologies, Wilmington, DE, USA).

Sequences covering the mutational hotspots in the TERT core promoter [nucleotide numbers 1,295,228 (C228T) and 1,295,250 (C250T) from the human reference sequence (GRCh37 February 2009; http://genome.ucsc.edu/)] were amplified by PCR. Primer sequences for the first PCR (Primer set 1) were 5′-GTC CTG CCC CTT CAC CTT-3′ (forward) and 5′-GCA CCT CGC GGT AGT GG-3′ (reverse).The first PCR was carried out using a C1000 thermal cycler (Bio-Rad, CA, USA) with an initial denaturing step at 95 °C for 3 min, followed by 40 cycles of denaturation at 96 °C for 15 s, annealing at 62 °C for 20 s, extension at 72 °C for 30 s, and a final extension at 72 °C for 10 min. Sequencing PCR: We used the SAP hydrolysed PCR products obtained by the first PCR as the template, and the primers were 5'-GTCCTGCCCCTTCACCTT-3' (reverse) (one-way sequencing). The amplification program was: 96 °C pre-denaturation for 60 s, followed by 30 cycles of denaturation at 96 °C for 10 s, annealing at 50 °C for 5 s, extension at 60 °C for 4min, and 4 °C constant temperature. Direct sequencing was performed with BigDye Terminator v3.1 (Applied Biosystems, Foster City, USA) on a sequencing instrument (ABI 3500Dx Genetic Analyzer, Applied Biosystems, Foster City, USA).

### Statistical analysis

Chi-square tests (Pearson's chi-square test, Correction for continuity) or Fisher's exact test was used in the analysis of correlations between the pathologic diagnosis and the parameters. Rank sum test (Mann Whitney U test) was used for ranked data. A multi-parameter diagnostic model for the pathological grade of T1 UBC was constructed by multivariable binary logistic regression analysis. All the above-mentioned statistical calculations were performed using SPSS 22.0 software (SPSS Inc., Chicago, IL, USA). Receiver operating characteristic (ROC) curve, the areas under the ROC curve (AUC), 95% confidence interval (CI), sensitivity, specificity, Youden's index, positive predictive value (PPV) and negative predictive value (NPV) of single marker and multi-parameter diagnostic model was produced using MedCalc v15.8 software. The AUCs were compared through Z test. P < 0.05 was considered statistically significant.

## Results

### Patient characteristics

There were 124 cases of bladder urothelial lesions diagnosed pathologically that had negative clinical examination margins, including 33 cases of non-cancerous lesions (11 cases of hyperplastic/glandular cystitis, 6 cases of chronic mucosal inflammation, 2 cases of papilloma, 7 cases of inverted papilloma and 7 cases of papillary urothelial neoplasm of low malignant potential). 106 cases were male and 18 cases were female. There were 78 cases with papillary growth and 46 cases with non-papillary growth. The number of lesions > 3 is 39 cases and ≤ 3 is 85 cases. There were 46 cases with lesion size ≥ 3 cm and 78 cases with lesion size < 3 cm (Table 2). There were 91 cases of UBC in T1 stage (28 cases of low grade and 63 cases of high grade). 82 cases were male and 9 cases were female in T1 UBC patients. The male to female ratio was 9.11:1 and the median age was 61 years old. There were 59 cases with papillary growth and 32 cases with non-papillary growth. The number of tumors > 3 is 32 cases and ≤ 3 is 59 cases. There were 39 cases with tumor size ≥ 3 cm and 52 cases with tumor size < 3 cm (Table 3).

### Association between parameters and urothelial lesions of bladder

The constituent ratios of male patients, lesion size ≥ 3 cm and MI > 2 in cancer patients (T1 UBC) were 90.1%, 42.9% and 45.1%, respectively, which were higher than those in non-cancer patients (72.7%, 21.2%, 3.0%, respectively) (P < 0.05). The positive rates of P53 and Ki-67 (+, >15%) in cancer patients were 58.2% and 62.6%, respectively, which were higher than those in non-cancer patients (24.2% and 3%, respectively) (P < 0.05). The expression of P504S protein was related to the urothelial lesions (P < 0.05). A significant difference was observed in CK20 abnormal expression between non cancer patients (6.1%) and cancer patients (96.7%) (P < 0.05). 56.0% of cancer patients and 6.1% of non-cancer patients had TERT promoter hot spot mutations (C228T and C250T) (Fig. 1E), the difference was statistically significant (P<0.05). Although the constituent ratios of age ≥ 70, number of lesions > 3 and papillary growth pattern in cancer patients was higher than that in non-cancer patients, the difference was not statistically significant (P > 0.05) (Table 2). In a word, gender, lesion size, MI, CK20, P53, Ki-67, P504S and TERT promoter hot spot mutations were correlated with the lesion properties (P < 0.05).

### Association between parameters and pathological grade of T1 UBC

In 91 cases of T1 UBC, 7.1% of low-grade cases and 61.9% of high-grade cases showed MI > 2 (P < 0.05). The positive rate of Ki-67 (+, >15%) in T1 UBC-HG (81.0%) was higher than those in T1 UBC-LG (21.4%) (P < 0.05). The expression of P504S protein was correlated with pathological grade (P < 0.05). Generally speaking, MI, Ki-67 and P504S were correlated with the pathological grade of T1 UBC (P < 0.05) (Table 3, Figure 1). However, age, gender, number and size of tumors, papillary growth pattern, CK20, P53 and TERT promoter hot spot mutations were not associated with the pathological grade of T1 UBC (P < 0.05).

### Multivariable binary logistic regression analysis and establishment of multi-parameter diagnostic model

Univariate analysis showed that the expression of MI, Ki67 and P504S were correlated with pathological grade of T1 UBC. The multi-parameter diagnostic model of the three parameters for the pathological grade of T1 UBC was constructed by multivariable binary logistic regression analysis, and the equation of multi-parameter diagnostic model was: In[p/(1-p)]=2.909×MI+2.638×Ki67+ 1.075×P504S_weak_-0.265×P504S_strong_-1.612 (χ^2^= 49.472; P < 0.05). The results showed that the risk of high-grade in patients with MI > 2 (+) was 18.331 times higher than that of MI ≤ 2 (-) [OR (95%CI) = 3.165-106.172; P <0.05]. The risk of high-grade in patients with Ki67 (+, >15%) was 13.989 times higher than that of Ki67 (-, ≤15%) (OR [95% CI)=3.691-53.022; P <0.05]. In brief, MI and Ki67 were independent risk factors for T1 UBC-HG (Table 4).

### Diagnostic value of multi-parameter diagnostic model in predicting pathological grade of T1 UBC

**Figure 2:**
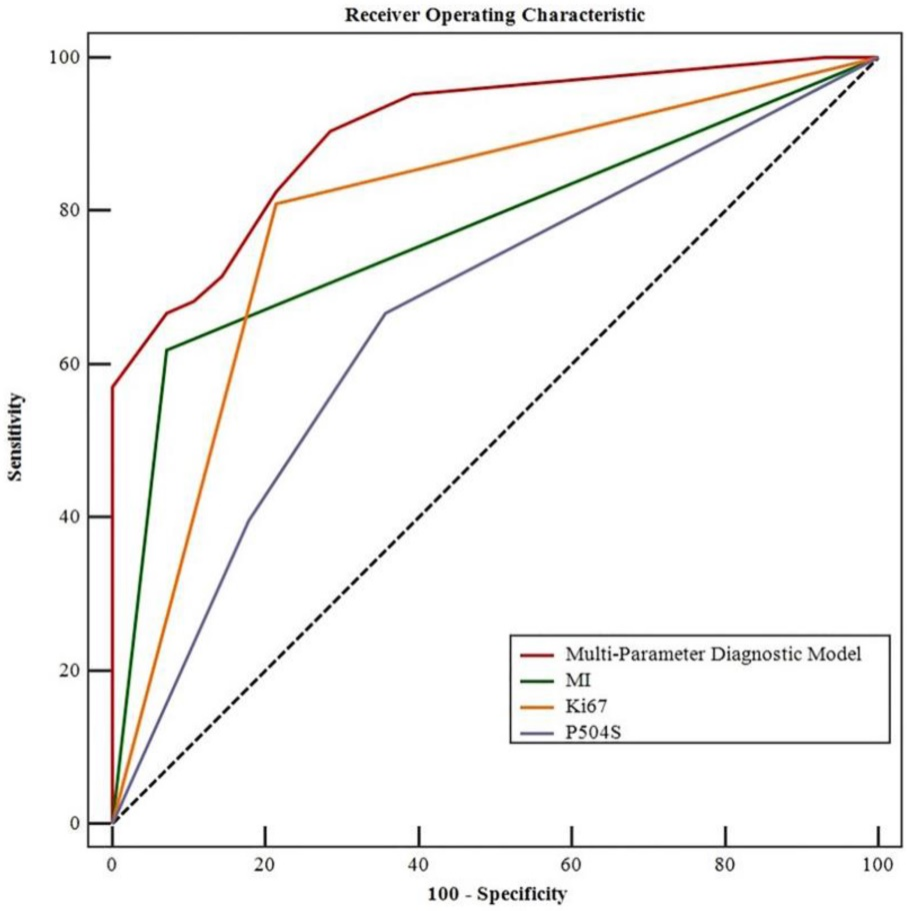
Multiparametric ROC curve related to the pathological grade of the T1 UBCs (MedCalc ROC analysis).

The ROC curve analysis of single MI, Ki-67, P504S and the combination in T1 UBC were plotted in Figure 2. The areas under the ROC curve (AUC) for the three markers in pathological grade of T1 UBC were: MI: 0.774; Ki-67: 0.798; P504S: 0.666. Among them, the AUC of Ki67 (AUC=0.798, 95%CI: 0.700~0.875, P<0.05) was the highest. Whereas, the AUC of multi-parameter diagnostic model of the three markers (AUC=0.904, 95%CI: 0.824~0.956, P<0.05) was higher than that of each marker detected respectively. The sensitivity was increased to 90.5% and the specificity was up to 71.4% when these three markers were combined. The corresponding positive predictive value (PPV) was 87.7%, and the negative predictive value (NPV) was 76.8%. The AUCs of multi-parameter diagnosis model and single detection of markers were compared through Z-test. Compared with the combined detection in T1 UBC, the Z values of MI, Ki-67 and P504S were 3.990, 2.886 and 4.392 respectively (P < 0.05). These results showed that there was a significant difference between combined detection and single marker detection of AUCs in T1 UBC (P < 0.01). In short, the multi-parameter diagnosis model of MI, Ki-67 and P504S diagnosed the pathological grade of T1 UBC better than single detection (Table 5, Figure 2).

## Discussion

Clinical decision and management of T1 UBC have been proven difficult due to the lack of reliable prognostic markers. The risk of recurrence and progression is often measured by the tumor grade of UBC. In pathological diagnosis, the grade of UBC is determined by the degree of architectural disorder and cytologic atypia 6, including cell density, polar direction, nuclear size, morphology, chromatin, nucleolus and MF. However, the reproducibility of this two-grade classification system is limited in regards to pathological diagnosis, and there is a lack of ideal, objective and easily detected markers for pathological diagnosis. A series of previous studies have suggested some common and valuable factors in the diagnosis and prognosis of UBC, but there are still some controversial factors. The purpose of this study was to evaluate the value of these factors in the pathological grading diagnosis of T1 UBC.

The grade of UBC is determined by the atypia of tissue structure and cells, and the reproducibility of pathological diagnosis is limited. The number and type of MF in tumors are very effective indicators for distinguishing between benign and malignant lesions, as well as suggesting the degree of malignancy and the prognosis 17. In the 2016 WHO classification, it is mentioned that MF is related to the classification of UBC, but there is no recommended threshold. Studies have shown that a high Ki-67 index and MF, as important markers of cell cycle activity, are closely related to the recurrence and progression of NMIBC 10, 18. Volume corrected mitotic index (mitotic figures/volume, M/V) is independent prognostic information for UBC, and the threshold of M/V is 10/mm^2^ 15-17. In our research, mitotic index (MI) is a quantitative mitotic frequency analysis measured by M/V index, which combines the number of mitotic figures with square millimeter (mm^2^) of neoplastic epithelium. We used a threshold of MI set to 2/HPF (field area: 0.2376 mm^2^) nearly 10/mm^2^, such as MI ≤ 2/HPF (- or mitotic-inert) and > 2/HPF (+ or mitotic-active). Although Ki-67 index were related to the pathological grade of T1 UBC, it has not yet been confirmed as a poor prognosis marker in NMIBC patients because the reported thresholds of positivity vary, making direct comparisons difficult 19. After literature review and statistical evaluation, we used the threshold of the Ki-67 set to 15%, such as Ki-67≤ 15% (- or low) and >15% (+ or high) 10, 20. We found that the MI and Ki-67 index were related to the pathological grade of T1 UBC. Mitotic-active (MI> 2/HPF) and high Ki-67 index (>15%) are effective markers of T1 UBC-HG. MI, as a morphological parameter, and Ki-67 index are straightforward and their diagnostic reproducibility is good, which may be beneficial for the accurate development of individualized treatment of UBC.

P504S, also known as alpha-Methylacyl-CoA racemase (AMACR), exists in mitochondria and peroxisomes and is involved in the β oxidation of fatty acids and fatty acid derivatives. At present, it is thought that P504S may be closely related to the occurrence and development of tumors 21, 22. However, it is not clear whether P504S is directly involved in the occurrence and progression of tumors or whether P504S is only an accompanying phenomenon in which tumor cells use alternative pathways to supply energy 23. The expression of P504S in upper tract urothelial carcinoma (UTUC) was studied. It was found that 127 cases were positive (positive rate 48.7%). The expression of AMACR/P504S was related to the stage and grade of the tumor, and thus, it may be a new prognostic marker for UTUC 23. A group of studies mainly aimed at non-invasive bladder tumors found that the expression of P504S was significantly correlated with the stage and grade of non-invasive bladder tumors 13. In our study, it was found that P504S was related to the pathological grade of T1 UBC, the multi parameter diagnosis model of MI, Ki-67 and P504S improved the diagnostic accuracy of assigning the T1 UBC pathological grade.

In the normal urinary epithelium, CK20 is only expressed in umbrella cells. Studies have shown that the abnormal expression of CK20 is a marker of urinary epithelial dedifferentiation and an early event of tumorigenesis 24. There are many reports supporting a prognostic role of CK20 expression in non-invasive urothelial tumors, with nonrecurrence rates varying from 45% to 100% in the presence of normal CK20 expression and the abnormal expression of CK20 was not related to the grade of UBC 24-27. In one study 28, multivariate Cox regression analysis revealed CK20 to be an independent prognostic factor for RFS (p=0.008) in stage pT1 UC, and the abnormal expression of CK20 was significantly correlated with worse cancer-specific survival and higher recurrence rates 10. In our study, it was found that CK20 was of great significance in the differential diagnosis of carcinomatous and non-cancerous lesions, but it was not related to T1 UBC grade. This observation may be due to the possibility of different CK20 staining patterns in urinary epithelial tumors, which makes it difficult to divide the subgroups of a disease using a single abnormal expression pattern of CK20.

The P53 tumor suppressor gene is a gene of the utmost importance to human tumors. P53 gene mutations occur frequently in UBC 29. The wild type P53 gene is a tumor suppressor gene. Mutations in P53 change its normal protein structure and decrease its suppressive function, resulting in the transformation of cells 30, 31. Mutant P53 protein can be detected quickly and accurately by immunohistochemistry. In our study, it was found that mutant P53 protein was related to the pathological diagnosis of T1 UBC but not to tumor grade. It proved again that the pathway involving mutant P53 was only one of the molecular pathways for the occurrence and development of UBC 32.

Telomerase activity imparts eukaryotic cells with unlimited proliferation capacity. Telomerase reverse transcriptase (TERT) is a key subunit of telomerase. Point mutations in the TERT promoter occur at a high frequency in multiple cancers, including urothelial cancer (UC). TERT activation is a fundamental step in tumorigenesis 33. The mutation rate of this promoter in urothelial tumors exceeds that of any other mutation, and it is widely present in various pathological grades and clinical stages 34. In bladder cancer, the mutations C228T and C250T are the most common 35. These promoter mutations in the urinary tract have tissue specificity in the occurrence of urothelial tumors, and these hot spot mutations have been reported in at least 55-83% of primary bladder cancers while only the wild-type promoter sequence was present in the 9 benign proliferative urothelial lesions 34. In our study, it was found that the hot spot mutations of TERT promoter were related to malignant urinary epithelial lesions, and they played an auxiliary role in the diagnosis of urothelial carcinoma, but they were of little significance in the diagnosis of UBC pathological grade.

This study shows that a combined multivariate (MI, Ki-67, P504S) diagnostic model can improve the diagnostic accuracy of the grading for T1 UBC, among which MI and Ki-67 have higher diagnostic values, which once again proves their significance in UBC pathology.

Regarding limitations, this study is a retrospective study. Although T1 UBC patients have been treated with bladder perfusion after TURBT, there is a little of inconsistency in the type of chemotherapeutic drugs and the choice of treatment cycle. Although more than 124 cases of paraffin samples were collected, some cases were not included in this study because some samples had DNA breaks and could not be detected for TERT promoter mutation. The above two aspects may have a certain impact on the research results.

## Conclusion

The results suggest that the MI, CK20, P53, Ki-67, P504S and TERT promoter hot spot mutations (C228T and C250T) can be meaningful markers in the diagnosis of UBC. In our research, we used a threshold of MI set to 2/HPF (0.2376 mm^2^), and the threshold of the Ki-67 was set to 15%. Patients with mitotic-active (MI>2/HPF) or/and high Ki-67 index had significantly higher pathological grade of the T1 UBC. Combined detection of the MI, Ki-67 index and P504S in multivariate diagnostic model can improve the diagnostic accuracy of assigning the T1 UBC pathological grade. MI and Ki-67, as important markers of histopathology and cell proliferation, are easily evaluated and show good reproducibility in diagnosis. In addition, these factors may be very meaningful parameters for assigning the pathological grade of UBC.

## Figures and Tables

**Table 1:** Positive expression threshold of each immunohistochemical marker

Parameter	Threshold	References
CK20	Normal expression: only seen in umbrella cells; Abnormal expression: diffuse staining (>5%) or completely missing	10
P504S	<5 % (-); >5 % (+); weak (1+); strong (2+)	13*
P53	≥10 % (+), <10 % (-)	10
Ki-67	>15 % (+ or high), ≤ 15 % (- or low)	10

Note: ^*^P504S positive staining was defined as having more than 5% of tumor cells showing diffuse cytoplasmic staining. This cut-off was chosen to exclude possible nonspecific and/or artificial staining. The staining intensity was graded as negative (0), weak (1+) and strong (2+).

**Table 2:** Association between parameters and urothelial lesions of bladder

Parameter	Property			
	Non-carcinoma (%)	Carcinoma (%)	χ^2^	*P* value
**Age**				
<70	26 (78.8)	62 (68.1)	1.335	0.248
≥70	7 (21.2)	29 (31.9)		
**Gender**				
Female	9 (27.3)	9 (9.9)	4.579	**0.032^&^**
Male	24 (72.7)	82 (90.1)		
**Number of lesions**				
Solitary (≤3)	26 (78.8)	59 (64.8)	2.187	0.139
Multiple (>3)	7 (21.2)	32 (35.2)		
**Lesion size**				
<3 cm	26 (78.8)	52 (57.1)	4.862	**0.027**
≥3 cm	7 (21.2)	39 (42.9)		
**Lesion shape**				
Papillary	19 (57.6)	59 (64.8)	0.547	0.460
Non-papillary	14 (42.4)	32 (35.2)		
**Mitotic index**				
≤2 (Mitotic-active)	32 (97.0)	50 (54.9)	19.095	**<0.001**
>2 (Mitotic-inert)	1 (3.0)	41 (45.1)		
**CK20**				
Normal	31 (93.9)	3 (3.3)	99.982	**<0.001**
Abnormal	2 (6.1)	88 (96.7)		
**P53**				
-	25 (75.8)	38 (41.8)	11.201	**0.001**
+	8 (24.2)	53 (58.2)		
**Ki67**				
- (Low)	32 (97.0)	34 (37.4)	34.562	**<0.001**
+ (High)	1 (3.0)	57 (62.6)		
**P504S**				
-(0)	20 (60.6)	39 (42.9)	-2.194	**0.028^#^**
Weak (1+)	11 (33.3)	30 (33.0)		
Strong (2+)	2 (6.1)	22 (24.2)		
**TERT promoter mutation**				
-	31 (93.9)	40 (44.0)	24.723	**<0.001**
+	2 (6.1)	51 (56.0)		

Note: ^&^Correction for continuity; ^#^Mann Whitney U test.

**Table 3:** Association between parameters and pathological grade of T1 UBC

Parameter	Grade			
	Low (%)	High (%)	χ^2^	*P* value
**Age**				
<70	22 (78.6)	40 (63.5)	2.030	0.154
≥70	6 (21.4)	23 (36.5)		
**Gender**				
Female	1 (3.6)	8 (12.7)	0.933	0.334^&^
Male	27 (96.4)	55 (87.3)		
**Number of tumors**				
Solitary (≤3)	19 (67.9)	40 (63.5)	0.162	0.687
Multiple (>3)	9 (32.1)	23 (36.5)		
**Tumor size**				
<3 cm	19 (67.9)	33 (52.4)	1.896	0.169
≥3 cm	9 (32.1)	30 (47.6)		
**Tumor shape**				
Papillary	19 (67.9)	40 (63.5)	0.162	0.687
Non-papillary	9 (32.1)	23 (36.5)		
**Mitotic index**				
≤2 (Mitotic-active)	26 (92.9)	24 (38.1)	23.482	**<0.001**
>2 (Mitotic-inert)	2 (7.1)	39 (61.9)		
**CK20**				
Normal	2 (7.1)	1 (1.6)		0.2323^*^
Abnormal	26 (92.9)	62 (98.4)		
**P53**				
-	14 (50.0)	24 (38.1)	1.130	0.288
+	14 (50.0)	39 (61.9)		
**Ki67**				
- (Low)	22 (78.6)	12 (19.0)	29.347	**<0.001**
+ (High)	6 (21.4)	51 (81.0)		
**P504S**				
- (0)	18 (64.3)	21 (33.3)	-2.331	**0.020^#^**
Weak (1+)	5 (17.9)	25 (39.7)		
Strong (2+)	5 (17.9)	17 (27.0)		
**TERT promoter mutation**				
-	14 (50.0)	26 (41.3)	0.600	0.439
+	14 (50.0)	37 (58.7)		

Notes: ^&^Correction for continuity; ^*^Fisher's exact test; ^#^Mann Whitney U test.

**Table 4:** Multivariable binary logistic regression analysis of T1 UBC pathological grade

Parameter	β	SE	Wald χ^2^	OR (95%CI)	*P* value
MI	2.909	0.896	10.534	18.331 (3.165~106.172)	**0.001**
Ki67	2.638	0.680	15.061	13.989 (3.691~53.022)	**<0.001**
P504S			2.517		0.284
P504S_weak_	1.075	0.783	1.887	2.930 (0.632~13.581)	0.170
P504S_strong_	-0.265	0.828	0.103	0.767 (0.151~3.884)	0.748
Constant	-1.612	0.554	8.452	0.200	0.004

**Table 5:** Parameters and multi-parameter diagnostic model for predicting the area under the curve (AUC) at T1 UBC pathological grade (MedCalc ROC analysis)

Parameter	Grade							
	AUC (95%CI)	Youden's index	Sensitivity (%)	Specificity (%)	PPV (%)	NPV (%)	Z value	*P* value
MI	0.774 (0.674- 0.855)	0.548	61.9	92.9	95.1	52.0	6.921	**<0.001**
Ki67	0.798 (0.700- 0.875)	0.566	81.0	78.6	89.5	64.7	6.373	**<0.001**
P504S	0.666 (0.560- 0.762)	0.310	66.7	64.3	80.8	46.2	2.904	**0.003**
Multi-Parameter Diagnostic Model	0.904 (0.824-0.956)	0.619	90.5	71.4	87.7	76.8	13.132	**<0.001**

## Abbreviations

T1 UBC: The stage T1 urothelial bladder cancer
UBC: urothelial bladder cancer
UC: urothelial cancer
TERT: telomerase reverse transcriptase
HG: high-grade
LG: low-grade
BCG: Bacille Calmette-Guerin
UICC/AJCC: Union for International Cancer Control/American Joint Committee on Cancer
MF: mitotic figure
MI: mitotic index
HE: hematoxylin-eosin staining
TURBT: transurethral resection of the bladder tumor
NCCN: National Comprehensive Cancer Network
ROC: Receiver operating characteristic
AUC: the areas under the ROC curve
PPV: positive predictive value
NPV: negative predictive value
AMACR: alpha-Methylacyl-CoA racemase
UTUC: upper tract urothelial carcinoma
CI: confidence interval
